# Papillary Thyroid Carcinoma Arising in Struma Ovarii With Subsequent Thyroid Microcarcinoma: A Case Report and Review of the Literature

**DOI:** 10.7759/cureus.82198

**Published:** 2025-04-13

**Authors:** Fernando Semanate, Sebastian Montoya, Esteban Andrade, Christian Palacios, Tatiana Fernandez Trokhimtchouk

**Affiliations:** 1 Surgical Oncology, Hospital de Especialidades Carlos Andrade Marín, Quito, ECU; 2 General Surgery, Universidad de las Américas, Quito, ECU

**Keywords:** malignant struma ovarii, metachronous tumor, ovarian teratoma, papillary thyroid carcinoma, struma ovarii, thyroidectomy, thyroid microcarcinoma

## Abstract

Struma ovarii is a rare form of monodermal ovarian teratoma composed predominantly of thyroid tissue. In exceptional cases, malignant transformation occurs, most commonly as papillary thyroid carcinoma. The subsequent appearance of thyroid malignancy in the cervical gland presents a diagnostic and therapeutic dilemma, particularly in differentiating between metastatic disease and metachronous or synchronous primary tumors. We report the case of a 43-year-old female patient with hypothyroidism who underwent laparoscopic adnexectomy for a symptomatic ovarian mass. Histopathological examination revealed a mature cystic teratoma with a focus of classic papillary thyroid carcinoma, without evidence of capsular or vascular invasion. Three years later, thyroid ultrasound identified a subcentimeter nodule classified as TIRADS 5, and fine-needle aspiration confirmed Bethesda VI cytology. Total thyroidectomy revealed a 4 mm unifocal classic papillary carcinoma, with no capsular or lymphovascular invasion and negative lymph nodes. Following thyroidectomy, no adjuvant radioactive iodine therapy was administered, and the patient remains disease-free after three years of surveillance. This case highlights the clinical challenge in distinguishing metachronous primary thyroid carcinoma from metastatic disease in patients with a prior diagnosis of malignant struma ovarii. Furthermore, it raises important considerations regarding the need for thyroidectomy and radioiodine therapy in such patients, especially when subcentimeter thyroid nodules are identified. In the absence of consensus guidelines, therapeutic decisions must be individualized, guided by tumor behavior, patient risk factors, and the available evidence. Reporting such cases is essential to inform clinical practice and refine follow-up strategies in this rare clinical scenario.

## Introduction

Struma ovarii (SO) is a rare monodermal ovarian teratoma predominantly composed of mature thyroid tissue, accounting for less than 1% of all ovarian tumors and approximately 2-3% of ovarian teratomas [1,2]. While the majority of SOs are benign, approximately 5% undergo malignant transformation, most commonly into papillary thyroid carcinoma (PTC), followed by follicular and, more rarely, poorly differentiated thyroid carcinoma [3,4]. Malignant struma ovarii (MSO) presents a diagnostic and therapeutic challenge due to its rarity, nonspecific clinical manifestations, and lack of standardized guidelines for postoperative management [5].

Patients with MSO are often asymptomatic or present with vague pelvic symptoms, and preoperative imaging frequently fails to distinguish between benign and malignant components [4,6]. Although surgical excision remains the primary treatment, controversies persist regarding the role of adjuvant total thyroidectomy and radioactive iodine (RAI) therapy, particularly in cases without extraovarian dissemination [5,7].

A unique subset of cases involves the subsequent diagnosis of thyroid malignancy in the cervical gland, raising a critical question: does this represent metastatic spread from the ovarian lesion, or an independent primary tumor? Metastasis of MSO to the thyroid is considered exceedingly rare, with few cases documented in the literature [8]. In contrast, the coexistence of synchronous or metachronous primary tumors, though rare, has been increasingly recognized [9,10]. Molecular studies have shown concordant BRAF V600E mutations in some of these cases, suggesting a shared pathogenesis, but definitive differentiation remains elusive without full molecular profiling [11].

In the context of incidentally discovered thyroid microcarcinomas, which are frequently managed with active surveillance, the presence of a prior MSO complicates the decision-making process. There are currently no evidence-based recommendations guiding the extent of treatment in these scenarios, and management often relies on individualized risk assessment.

Herein, we present the case of a 43-year-old woman with classic PTC arising within a struma ovarii, who was later diagnosed with a thyroid microcarcinoma during surveillance. Through this report, we aim to review the available evidence and highlight the clinical dilemmas in diagnosis, classification, and management of patients with dual thyroid-type malignancies.

## Case presentation

**Figure 1:**
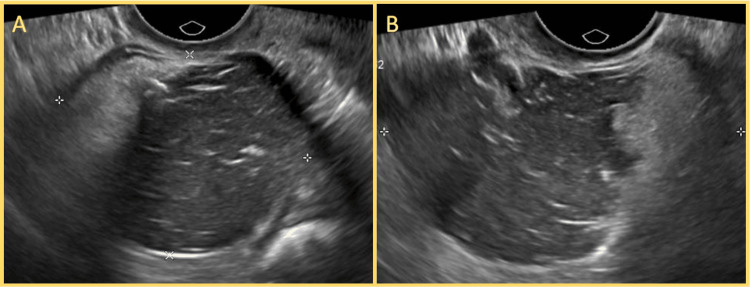
Transvaginal ultrasound of the left adnexal mass (A, B) Longitudinal views showing a complex cystic lesion in the left ovary, approximately 3 cm in diameter. The mass exhibits heterogeneous internal echoes, thin septations, and irregular walls. These sonographic features were suggestive of a complex ovarian cyst and prompted further evaluation by laparoscopy and left adnexectomy.

A 43-year-old woman with a history of hypothyroidism, well-controlled on levothyroxine therapy (thyroid-stimulating hormone (TSH) within normal range), presented five years prior to the gynecology service with complaints of lower abdominal pain and dyspareunia. Pelvic ultrasound revealed a 3 cm complex cystic lesion in the left ovary (Figure 1). She underwent diagnostic laparoscopy with left adnexectomy. The immediate postoperative course was uneventful, and the patient was discharged within 24 hours.

Histopathological examination of the surgical specimen revealed a mature cystic teratoma composed predominantly of thyroid tissue. Embedded within the teratomatous tissue was a focus of malignant epithelial proliferation consistent with papillary thyroid carcinoma, classic variant. No capsular, lymphovascular, or perineural invasion was observed. The patient was subsequently referred to the clinical oncology service for surveillance.

During follow-up, she experienced an anembryonic pregnancy that required cervical preparation. One year later, she conceived again and underwent cesarean section at 38.6 weeks due to cephalopelvic disproportion, with an uneventful postpartum recovery. Her hypothyroidism worsened during pregnancy, requiring levothyroxine dose adjustment, which was later stabilized.

**Figure 2:**
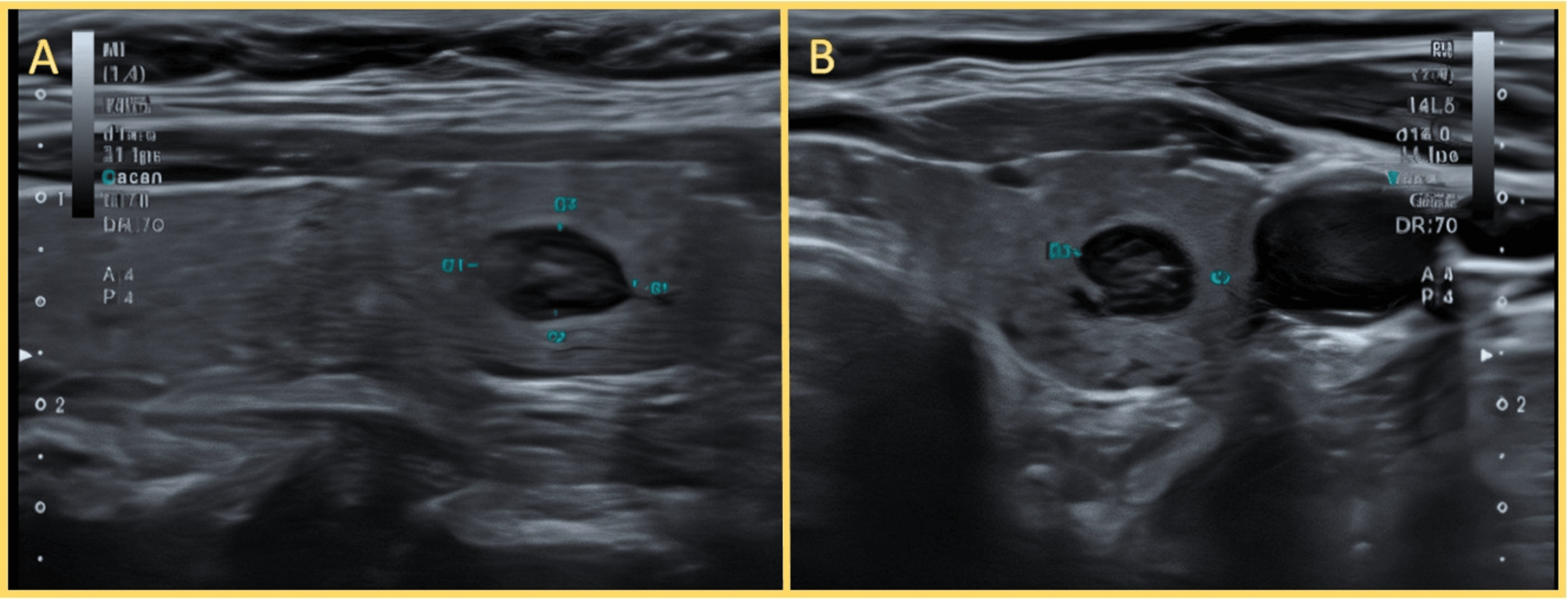
Thyroid ultrasound showing suspicious nodule in the left lobe (A, B) Transverse views of the left thyroid lobe demonstrating a hypoechoic, solid nodule with irregular margins and taller-than-wide orientation. The lesion measures approximately 0.5 cm and was classified as TIRADS 5 due to its ultrasonographic features. Fine-needle aspiration yielded Bethesda category VI cytology, confirming papillary thyroid carcinoma.

Approximately one year after delivery, a routine thyroid ultrasound revealed three nodules in the left lobe. The most concerning lesion measured 0.5 cm, was hypoechoic and heterogeneous with irregular margins, and was classified as TIRADS 5 (Figure 2). Fine-needle aspiration cytology of this nodule was consistent with Bethesda category VI. She was then referred to our Head and Neck Surgery Department.

**Figure 3:**
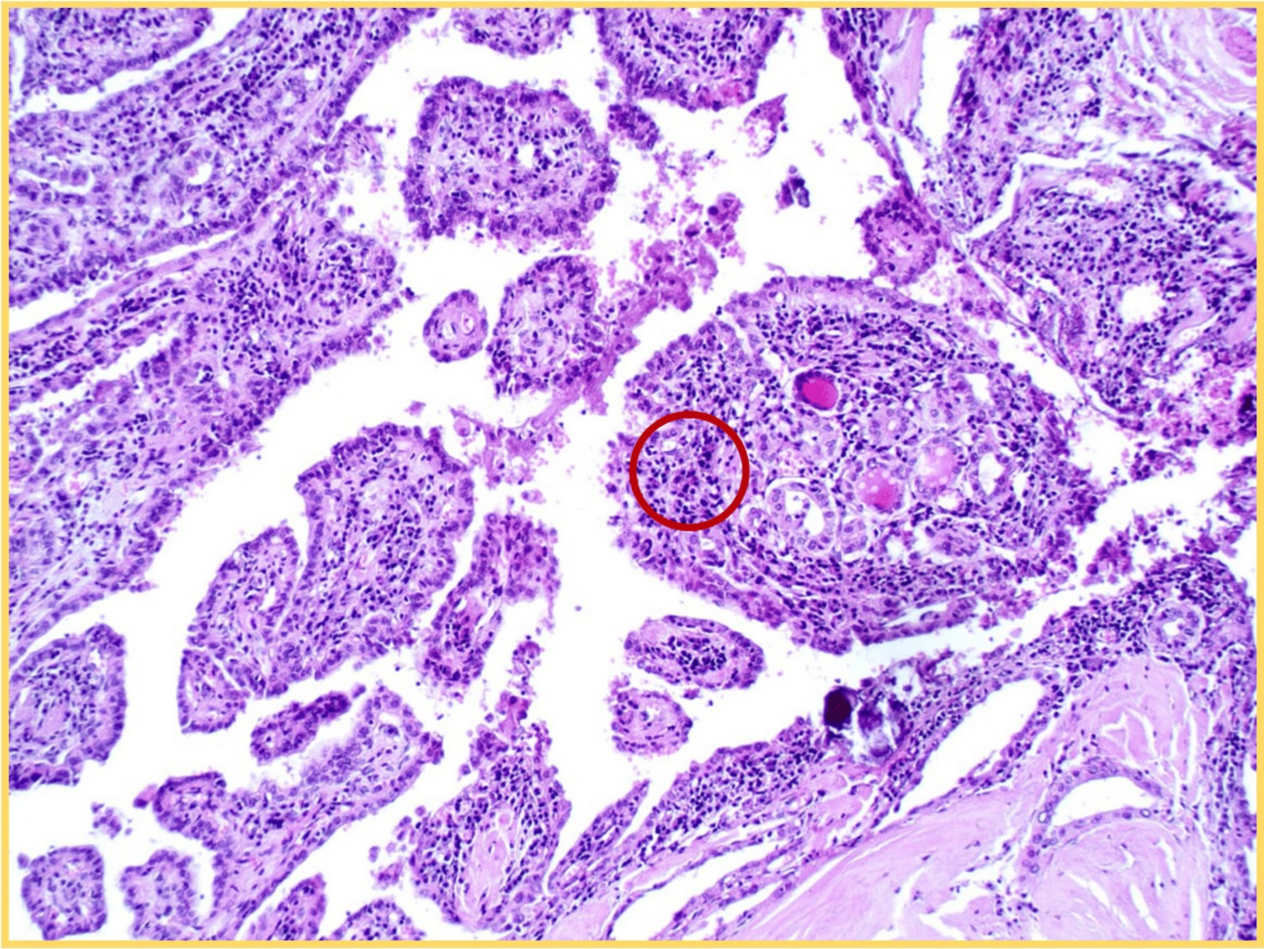
Histological section of the thyroid gland (H&E, original magnification ×100) The image demonstrates papillary structures with fibrovascular cores. Some nuclei exhibit features suggestive of papillary thyroid carcinoma, including nuclear crowding and overlapping (highlighted in the red circle). These findings support the diagnosis of classical papillary thyroid carcinoma. Image interpretation provided by Dr. Johnny Torres Parrales, Department of Pathology.

The patient underwent total thyroidectomy without complications. Final histopathology confirmed a unifocal classic papillary thyroid carcinoma measuring 4 mm, confined to the left lobe, without capsular or lymphovascular invasion (Figure 3). One incidental lymph node retrieved with the specimen was negative for malignancy. Based on the low-risk profile and in accordance with current guidelines, adjuvant radioactive iodine (RAI) therapy was not administered. 

The patient has been under our surveillance for the past three years, with periodic ultrasound, TSH, and serum thyroglobulin assessments, all of which have shown no evidence of recurrence to date.

## Discussion

MSO is a rare entity representing a minority of ovarian teratomas, with PTC being the most common histologic subtype [3,4]. Its clinical presentation is frequently nonspecific, and preoperative imaging often fails to differentiate benign from malignant lesions [4,6]. In some cases, hyperthyroidism may be present, although it occurs in fewer than 10% of patients [2]. 

Definitive diagnosis is histopathological and tipically incidental. Due to the rarity of MSO, large-scale prospective studies are lacking, and management is primarily guided by case reports, small retrospective series, and expert consensus. Surgical resection continues to be the cornerstone of treatment, with salpingo-oophorectomy favored over ovarian cystectomy given its lower recurrence risk [4]. While fertility-sparing surgery may be appropriate in selected cases, oophorectomy with or without hysterectomy is generally preferred in women who have completed childbearing [4,6]. 

Prognosis is generally favorable. In a cohort of 68 patients, Goffredo et al. reported 5-, 10-, and 20-year overall survival (OS) rates of 96.7%, 94.3%, and 84.9%, respectively [10]. However, the presence of aggressive histologic subtypes or extraovarian spread is associated with worse outcomes [5,7].

The occurrence of a second thyroid malignancy following a diagnosis of MSO introduces a complex diagnostic dilemma. Distinguishing between metastatic disease, a synchronous malignancy, or a metachronous primary thyroid carcinoma is clinically significant, as each implies a different biological behavior, staging classification, and therapeutic approach.

Metastasis of MSO to the thyroid gland itself is exceedingly rare. Documented metastatic sites more commonly include lungs, bones, liver, and brain, following a hematogenous dissemination pattern rather than lymphatic spread to the cervical thyroid [8]. Well-documented cases of thyroidal metastasis from MSO are exceptional, making this a highly unlikely scenario in most patients.

Conversely, the coexistence of synchronous or metachronous thyroid carcinoma has been increasingly reported. In the same study by Goffredo et al., 8.8% of MSO patients were diagnosed with a primary thyroid carcinoma either concurrently or during follow-up [10]. Similarly, Sisti et al. described two synchronous and one metachronous case in a series of 21 patients [9]. Although these data are limited, they underscore the importance of long-term surveillance in patients with MSO.

Histologically, the presence of similar architectural and cytologic features in both lesions-such as the classic variant of PTC-does not confirm a clonal relationship. Molecular profiling, particularly the identification of shared mutations like BRAF V600E, has suggested a possible common origin in some synchronous cases [11]. However, these findings are not consistent across all reports, and in the absence of such analysis, differentiation must rely on temporal, clinical, and radiological criteria.

In our case, the absence of extraovarian spread at initial diagnosis, the small size and confinement of the thyroid lesion, and the three-year disease-free interval strongly support the diagnosis of a metachronous primary thyroid carcinoma rather than metastatic disease. Nonetheless, the histologic similarity between the two tumors illustrates the ongoing diagnostic uncertainty clinicians face in these scenarios.

The role of prophylactic total thyroidectomy following the diagnosis of MSO remains controversial. In patients with disease confined to the ovary and no evidence of metastasis or aggressive features, routine thyroidectomy is not universally indicated. The rationale for its use is twofold: to exclude an occult cervical thyroid primary, and to enable RAI therapy and thyroglobulin-based surveillance if needed.

However, evidence supporting this strategy is limited. Li et al., in a retrospective study of patients with MSO confined to the ovary, found no significant improvement in recurrence-free survival (RFS) in those who underwent thyroidectomy compared to those who did not. [5]. Similarly, Addley et al. emphasized the importance of individualized management based on tumor behavior, patient factors, and follow-up findings, rather than routine surgical intervention [6].

Despite this, some authors advocate for total thyroidectomy and RAI in all cases of MSO, particularly when tumors display aggressive histologic features, extraovarian extension, or when long-term biochemical monitoring is desired [4,6]. In the absence of such factors, a more conservative approach may be appropriate.

In the present case, the patient did not undergo thyroidectomy immediately after the diagnosis of MSO, which was confined to the ovary and lacked capsular or vascular invasion. This approach aligns with the current tendency toward individualized management and reflects the absence of robust data justifying routine thyroidectomy in low-risk cases.

However, when a suspicious thyroid nodule was identified during follow-up, diagnostic workup confirmed a second PTC. At that stage, surgical management followed standard thyroid cancer guidelines and was not based solely on the prior diagnosis of MSO. This sequence highlights the value of long-term, structured surveillance, and supports the notion that thyroidectomy should be considered in response to specific clinical or radiological findings-not as a routine measure.

Active surveillance has recently gained acceptance as a valid alternative to immediate surgery for low-risk papillary thyroid microcarcinoma (micro-PTC), defined as intrathyroidal tumors ≤1 cm without lymph node involvement or extrathyroidal extension [12,13]. Originally developed in Japan, this approach is now endorsed by international guidelines and supported by long-term data demonstrating excellent disease-specific survival and low progression rates in appropriately selected patients [12].

Nevertheless, in patients with a history of MSO, the decision to observe a newly discovered thyroid microcarcinoma becomes more nuanced. Although the thyroid lesion in this case-measuring <5 mm, unifocal, and intrathyroidal with no lymphovascular invasion-would typically qualify for active surveillance, the history of prior malignant thyroid-type neoplasm, albeit extra-thyroidal, complicates risk stratification. Theoretically, shared molecular drivers may be involved, although this remains speculative in the absence of confirmatory data.

Some authors have argued that patients with MSO should undergo thyroidectomy precisely to identify and treat potential occult thyroid primaries [4]. However, this strategy assumes a risk profile that may not be applicable in all cases. In the absence of adverse features on imaging or cytology, and without standardized molecular markers to guide management, such decisions must be individualized.

In the case reported here, the thyroid nodule was initially identified during surveillance, and although small and subcentimeter, it was classified as TIRADS 5 and yielded a Bethesda VI cytology on fine-needle aspiration. These findings, along with the history of MSO, justified proceeding with total thyroidectomy. Interestingly, the final pathology confirmed a micro-PTC that would otherwise meet criteria for active surveillance. This raises an important clinical question: would observation have been safe in this context?

At present, no clear consensus exists on how a history of MSO should influence management decisions in patients with incidentally discovered micro-PTC. Until better prognostic markers become available, clinical judgment remains paramount. In this case, the decision to proceed with thyroidectomy was not based solely on tumor size, but on the combination of cytologic suspicion, imaging findings, and oncologic history.

This case exemplifies the clinical complexity associated with MSO and subsequent thyroid neoplasia, highlighting the importance of individualized management in the absence of robust guidelines. It also illustrates how evolving paradigms, such as active surveillance, must be carefully adapted when patients fall outside the classical low-risk categories due to unique oncologic histories. Continued reporting of such cases will help refine diagnostic criteria and therapeutic algorithms for this rare but clinically significant scenario.

## Conclusions

Malignant struma ovarii is a rare neoplasm with predominantly favorable outcomes when confined to the ovary, but its coexistence with or progression to thyroid carcinoma poses significant diagnostic and therapeutic challenges. Differentiating between metastatic disease and metachronous or synchronous primaries remains complex, particularly in the absence of molecular profiling.

Our case underscores the importance of long-term surveillance in patients with MSO and illustrates how standard management paradigms, such as active surveillance for thyroid microcarcinoma, must be cautiously reconsidered in light of prior oncologic history.

In the absence of consensus guidelines, clinical judgment, close follow-up, and individualized decision-making are essential to optimize outcomes in these rare and complex clinical scenarios.
